# Evaluating the harmonisation potential of diverse cohort datasets

**DOI:** 10.1007/s10654-023-00997-3

**Published:** 2023-04-26

**Authors:** Sarah Bauermeister, Mukta Phatak, Kelly Sparks, Lana Sargent, Michael Griswold, Caitlin McHugh, Mike Nalls, Simon Young, Joshua Bauermeister, Paul Elliott, Andrew Steptoe, David Porteous, Carole Dufouil, John Gallacher

**Affiliations:** 1 Dementias Platform UK, Oxford, UK; 2 Alzheimer Disease Data Initiative, Kirkland, Washington USA; 3 Evaluserve, Bengaluru, India; 4 National Institute of Aging, Bethesda, USA; 5 University of Mississippi, Oxford, USA; 6 Data Tecnica International LLC, Washington, USA; 7 Imperial College, London, England; 8 University College London, London, England; 9 University of Edinburgh, Edinburgh, Scotland; 10 University of Bordeaux, Bordeaux, France

**Keywords:** Data harmonisation, Cohort, Data visualisation, C-surv data model, Data discovery, Datasets

## Abstract

Data discovery, the ability to find datasets relevant to an analysis, increases scientific opportunity, improves rigour and accelerates activity. Rapid growth in the depth, breadth, quantity and availability of data provides unprecedented opportunities and challenges for data discovery. A potential tool for increasing the efficiency of data discovery, particularly across multiple datasets is data harmonisation.A set of 124 variables, identified as being of broad interest to neurodegeneration, were harmonised using the C-Surv data model. Harmonisation strategies used were simple calibration, algorithmic transformation and standardisation to the Z-distribution. Widely used data conventions, optimised for inclusiveness rather than aetiological precision, were used as harmonisation rules. The harmonisation scheme was applied to data from four diverse population cohorts.Of the 120 variables that were found in the datasets, correspondence between the harmonised data schema and cohort-specific data models was complete or close for 111 (93%). For the remainder, harmonisation was possible with a marginal a loss of granularity.Although harmonisation is not an exact science, sufficient comparability across datasets was achieved to enable data discovery with relatively little loss of informativeness. This provides a basis for further work extending harmonisation to a larger variable list, applying the harmonisation to further datasets, and incentivising the development of data discovery tools.

**Supplementary Information:**

The online version contains supplementary material available at 10.1007/s10654-023-00997-3.

## Background

Data discovery, the ability to find data assets relevant to an analysis, is a critical component of a productive research environment. Efficient data discovery increases opportunity, improves rigour, and accelerates activity. For cohort studies, the complexity and variety of longitudinal data presents particular discovery challenges as data structures and labelling conventions are highly variable and typically under-documented. The growth of data repositories [1–5] providing global 3rd party researcher access to multiple datasets, increases the value of efficient data discovery.

A pre-requisite for efficient data discovery is harmonisation. The goal of harmonisation is to achieve comparability (inferential equivalence) between two or more variables by inferring a latent construct that the variables are considered to represent. For example, different reaction time tasks may be judged to represent a latent construct of cognitive processing speed. However, latent constructs are purpose-specific, as a latent construct of processing speed based on simple reaction time may be inadequate for a hypothesis based on choice reaction time. Harmonisation is easily conflated with standardisation where data are organised (structured and annotated) according to a standard model. Clearly, standardisation is a step towards harmonisation, but they are not coterminous. Standardisation is about conformability whilst harmonisation is about comparability.

Previous exercises in the harmonisation of cohort data have focussed on hypothesis-specific testing across multiple datasets. The Maelstrom consortium has adopted a different approach by developing multiple harmonised datasets available for 3rd party use [6]. The Integrative Analysis of Longitudinal Studies of Aging (IALSA) consortium also supports a broad range of multi-cohort analyses; making the harmonisation code available for many of them [7]. The CLOSER consortium provides an extensive programme of topic-specific harmonisation initiatives across 19 cohorts [8], designed to test a broad range of hypotheses. Other initiatives are more focussed. Adhikari et al. describe harmonising 20 variables from two pregnancy cohorts to better understand risk factors for pre-term birth [9], whilst Almeida et al. describe and validate a pipeline for neurodegeneration analyses, also using two population cohorts [10]. Further larger harmonisation collaborative projects include the Research Advancement through Cohort Cataloguing and Harmonization (ReACH) [11], the EU Child Cohort Network [12] and the Melbourne Children’s LifeCourse (LifeCourse) [13] initiatives.

Here we focus on harmonisation for data discovery. Focussing on data discovery requires fewer assumptions about likely future use of the harmonised data, enabling the use of more generic latent constructs. It is also sympathetic to relatively simple harmonisation rules. Value lies in enabling the availability of relevant data from multiple datasets, to be assessed accurately and efficiently, prior to an access request. This reduces search costs for data discovery and reduces the risk of accessing and processing uninformative data. The work was conducted within the Dementias Platform UK Data Portal (DPUK) [1]. This is an integrated research environment for bona fide researchers interested in accessing cohort data for secondary analysis. The Portal provides tools for data discovery, an access management system and a virtual desktop interface (VDI) for analysis. The VDI provides a virtual desktop with preinstalled statistical programmes (e.g., Stata, Python, SPSS, R, MATLAB) for analysing complex multi-modal datasets. The data discovery tools (Cohort Matrix, Cohort Directory, Cohort Explorer) provide researchers with the ability to investigate appropriate cohorts at increasing levels of granularity. The Cohort Explorer (https://portal.dementiasplatform.uk/CohortExplorer) is premised on the harmonisation of a select number of 30 variables focused on neurodegeneration.

To evaluate the harmonisation potential of population cohort data for data discovery, members of the Alzheimer’s Disease Data Initiative (ADDI) [4] and DPUK formed a Data Harmonisation Group, to attempted the harmonisation of a comprehensive set (124 variables) of neurodegeneration related variables, across four diverse population cohort datasets. The aim of this project is to inform the development of discovery tools across the ADDI Workbench and the DPUK Data Portal.

## Methods

### Variable selection

A set of 124 variables optimised for neurodegeneration was identified by consensus within the ADDI Data Harmonisation group. Variables were selected to reflect the frequency of being requested in Dementias Platform UK (DPUK) data access proposals [1], to cover a range of data modalities, and to include modifiable and non-modifiable risk factors.

### Standardisation

Datasets were curated to a common structure and labelling conventions using C-Surv as the data model [14]. C-Surv is a simple four level acyclic taxonomy intended to capture the breadth of data typically collected in research cohorts. The tiered structure supports grouped and individual variable discovery. C-Surv comprises 18 data themes (level 1) leading to > 146 data ‘domains’ (level 2), > 500 data ‘families’ (level 3) and then to a growing number of data ‘objects’ (level 4) i.e. the measured variable. C-Surv has been adopted by DPUK [1], Dementias Platform Australia [3], and the ADDI workbench [4]. Other models, developed for other purposes were available, such as the Observational Health Data Sciences and Informatics (OHDSI) OMOP Common Data Model for administrative health data [15], and CDISC Clinical Data Interchange Standards Consortium (CDISC) for trials data [16], but these have structural and semantic complexity that is alien to the cohort study design.

### Schema development

The harmonisation schema was optimised to be inclusive of datasets by using relatively simple harmonisation rules and widely used value-labelling conventions. Three strategies for harmonisation, as described in the Maelstrom harmonisation guidelines [17] were used.

Simple calibration, using direct mapping between the source variable and the harmonised variable, was adopted for widely used standard metrics such as weight or height. Direct mapping, including cut-off points was used for validated clinical scales. The Gregorian calendar was used for dates and conventional units were used for age (years), durations (hours), concentrations (mg/ml), volumes (mm^3^), etc.

Algorithmic transformation was used for non-clinical questionnaire responses including lifestyle. The algorithm was selected to be inclusive by using a relatively simple transformation and was developed iteratively as it was applied to each dataset. Gender was transformed as male, female; smoking as ‘current, past, and never’, and ethnicity as white, black, Asian, mixed, other. Cohabitation was coded as single, married/cohabiting, divorced/separated, widowed, whilst education was considered as educational experience and transformed into junior or less, secondary, degree or equivalent, postgraduate or equivalent. For type of accommodation a straightforward transformation was house/bungalow, apartment, sheltered/residential, other.

Non-clinical cognitive performance scores were standardised into z-scores by default, with an option for refining this rule on a scale-by-scale basis according to the variable distribution. More sophisticated methods such as latent variable modelling or multiple imputation were not used.

### Schema evaluation

The utility of the harmonisation rules was tested using four DPUK collaborating cohorts. These were selected on the basis of having diverse primary scientific objectives, providing longitudinal multimodal data, and being frequently requested by DPUK users. The cohorts were the Airwave Health Monitoring Study (Airwave); an occupational cohort [18], the English Longitudinal Study of Ageing (ELSA); a social science focussed study [19], Generation Scotland; a genetics cohort [20], and Memento; a neurodegeneration cohort [21]. The coverage of each cohort and overlap of variables across cohorts was assessed, along with the utility of the harmonisation rules. All analyses were conducted within the DPUK Data Portal [22].

## Results

### Core variables

The variable list comprised a range of modifiable and non-modifiable risk factors and metadata (Table 1). Of the 124 variables, most variables (n = 103) were present in the baseline data. However, for ELSA data, 18 variables were collected in subsequent waves. For Memento, two outcomes were collected through linkage to health records. For both ELSA and Memento, genetics data are available independently of study wave. The variables covered 15 out of the 18 data themes represented by C-Surv data model (Table 1). Themes not represented were linkage data (theme 14), healthcare utilisation data (theme 15), and device data (theme 18).

**Table 1:** Core variable list

#	C-Surv theme	Variable	Strategy	Harmonisation rule
1	Administration:theme 1	Cohort ID	SC	Anonymised by cohort
2		Assessment date	SC	Gregorian calendar (yyyy-mm-dd)
3		Date of birth	SC	Gregorian calendar (yyyy-mm-dd)
4		Date of death	SC	Gregorian calendar (yyyy-mm-dd)
5		Cause of death	SC: text	ICD-11 categories 1–18
6		DNA extracted	SC	1 Yes; 0 No
7		Plasma collected	SC	1 Yes; 0 No
8		Serum collected	SC	1 Yes; 0 No
9		CSF collected	SC	1 Yes; 0 No
10	Sociodemographic:theme 2	Age	SC	Value: years 1–130
11		Gender	SC	1 male; 2 female
12		Ethnicity	AT	1 white; 2 Black; 3 Asian; 4 mixed 5 other
13		Cohabitation	AT	1 single; 2 married/cohabiting; 3 separated/divorced;4 widowed other
14		Years education	SC	Value: years range
15		Educational level	AT	1 postgrad; 2 degree; 3 secondary; 4 junior or less
16		Income	AT	Quantiles using local currency
17	Early life experience:theme 3	Childhood physical abuse	SC	1 Yes; 0 No
18		Adolescent physical abuse	SC	1 Yes; 0 No
19		Sexual abuse	SC	1 Yes; 0 No
20		Parental smoking behaviour	SC	1 Yes; 0 No
21	Medical history:theme 4	Type 1 diabetes diagnosis	AT	1 Yes; 0 No
22		Type 2 diabetes diagnosis	AT	1 Yes; 0 No
23		AD diagnosis	AT	1 Yes; 0 No
24		AD FTD diagnosis	AT	1 Yes; 0 No
25		AD mixed diagnosis	AT	1 Yes; 0 No
26		VaD diagnosis	AT	1 Yes; 0 No
27		PD diagnosis	AT	1 Yes; 0 No
28		Depression diagnosis	AT	1 Yes; 0 No
29		Self-report visual difficulty	AT	1 Yes; 0 No
30		Self-report hearing difficulty	AT	1 Yes; 0 No
31		Angina diagnosis	AT	1 Yes; 0 No
32		MI diagnosis	AT	1 Yes; 0 No
33		Hypertension diagnosis	AT	1 Yes; 0 No
34		Stroke diagnosis	AT	1 Yes; 0 No
35		Head injury	AT	1 Yes; 0 No
36		COPD diagnosis	AT	1 Yes; 0 No
37		Arthritis diagnosis	AT	1 Yes; 0 No
38		Current pain	AT	1 Yes; 0 No
39		Self-report general health	AT	1 Yes; 0 No
40		Medications	SC: text	Value: number prescribed
41	Family disease history: theme 5	Dementia parent	SC	1 Yes; 0 No
42		Dementia grandparent	SC	1 Yes; 0 No
43		Dementia sibling	SC	1 Yes; 0 No
44		AD parent	SC	1 Yes; 0 No
45		AD grandparent	SC	1 Yes; 0 No
46		AD sibling	SC	1 Yes; 0 No
47		VaD parent	SC	1 Yes; 0 No
48		VaD grandparent	SC	1 Yes; 0 No
49		VaD sibling	SC	1 Yes; 0 No
50		PD parent	SC	1 Yes; 0 No
51		PD grandparent	SC	1 Yes; 0 No
52		PD sibling	SC	1 Yes; 0 No
53		CHD parent	SC	1 Yes; 0 No
54		CHD grandparent	SC	1 Yes; 0 No
55		CHD sibling	SC	1 Yes; 0 No
56		Stroke parent	SC	1 Yes; 0 No
57		Stroke grandparent	SC	1 Yes; 0 No
58		Stroke sibling	SC	1 Yes; 0 No
59	Psychological status: theme 6	GHQ score	AT	Scale score
60		Self-report depression	AT	1 Yes; 0 No
61		Loss of interest	AT	1 Yes; 0 No
62		Depression score	AT	Scale score
63		EPQ Neuroticism	AT	Scale score
64		EQP Extraversion	AT	Scale score
65		Life satisfaction score	AT	Scale score
66		Job satisfaction score	AT	Scale score
67		Quality of Life score	AT	Scale score
68		Loneliness scale score	AT	Scale score
69	Cognitive status:theme 7	Immediate recall score	S	Z score
70		Delayed recall score	S	Z score
71		Digit symbol substitution score	S	Z score
72		Verbal fluency score	S	Z score
73		Choice reaction time mSec	S	Z score
74		Fluid intelligence score	S	Z score
75		MMSE score	SC	Scale score
76		ADAS cog total score	SC	Scale score
77		CDR total score	SC	Scale score
78		Subjective memory complaint	AT	1 Yes; 0 No
79		MCI diagnosis	AT	1 Yes; 0 No
80	Lifestyle: theme 8	Alcohol consumption	AT	Alcohol units per week, other
81		Smoking status	AT	0 never smoked; 1 past smoker; 2 current
82		Vigorous exercise	AT	1 Yes; 0 No
83		Moderate exercise	AT	1 Yes; 0 No
84		Walking	AT	1 Yes; 0 No
85		Sleep quality scale	AT	Scale score
86		Sleep hours per night	SC	Hours per night
87	Life functionality: theme 9	ADL score	AT	Scale score (higher value higher independence)
88		IADL score	AT	Scale score (higher value higher functioning)
89	Physical environment: theme 10	Number of house occupants	SC	Value (occupants)
90		Number of rooms	SC	Value (rooms)
91		Type of accommodation	AT	1 house/bungalow, 2 apartment, 3 residential/sheltered/ other
92		Pollution (grime in house)	SC	1 Yes; 0 No
93	Social environment: theme 11	Number of contacts/month	SC	Value (number of social contacts)
94		Social media sites used	SC	Value (number of sites used)
95		Social media use daily	SC	Value (types used daily)
96	Physical examination: theme 12	Height	SC	Value (cm)
97		Weight	SC	Value (kg)
98		BMI	SC	Value (ratio m^2^/kg)
99		Grip strength	SC	Value (kg)
100		Gait (walking) speed	SC	Value (m/sec)
101		Systolic BP	SC	Value (mm/hg)
102		Diastolic BP	SC	Value (mm/hg)
103	Imaging: theme 13	White matter volume	SC	Value (mm^3^ standardised)
104		Grey matter volume	SC	Value (mm^3^ standardised)
105		Left hippocampal volume	SC	Value (mm^3^ standardised)
106		Right hippocampal volume	SC	Value (mm^3^ standardised)
107		WM hyperintensities	SC	Value (mm^3^ standardised)
108		Amyloid PiB SUVR	SC	Ratio
109	Biosample assays: theme 16	Haemoglobin	SC	Value (mg/dl)
110		White cell count	SC	Value (mg/dl)
111		RBC count	SC	Value (mg/dl)
112		Total cholesterol	SC	Value (mg/dl)
113		HDL cholesterol	SC	Value (mg/dl)
114		Creatinine	SC	Value (mg/dl)
115		Glucose	SC	Value (mg/dl)
116		CRP	SC	Value (mg/dl)
117		Cortisol decrease	SC	Value (mg/dl)
118		Abeta 1–42	SC	Value (pg/ml)
119		Abeta 1–40	SC	Value (pg/ml)
120		Abeta 1–42	SC	Value (pg/ml)
121		Abeta 1–40	SC	Value (pg/ml)
122		Total tau	SC	Value (pg/ml)
123		P tau	SC	Value (pg/ml)
124	Molecular: theme 17	APOE status	SC	1 2/2; 2 2/3; 3 2/4; 4 3/3; 5 3/4; 6 4/4)

*SC* Simple calibration, *AT* Algorithmic transformation, *S* Standardisation

C-Surv themes not represented: Linkage data (theme 14), Healthcare utilisation data (theme 15), and Device data (theme 18)

### Representation and distribution

Most variables (n = 120; 97%) were found in one or more cohorts. Memento, being primarily designed to investigate neurodegeneration, included most variables (n = 92). The other cohorts, designed to address a broader range of questions had fewer neurodegeneration-focused variables (Table 2). Of the 4 variables that were not found in any cohort, one was related to air pollution (pm_2.5_ concentration) and another was loneliness assessment. That Mild Cognitive Impairment (MCI) status was not available in any cohort reflects the difficulty of capturing these data in a population setting. That ADAS-Cog score was not available reflects the use of this scale primarily in trials than in cohorts.

**Table 2:** Distribution of core variables across cohorts

Harmonised dataset		Number of variables per cohort			
C-Surv theme	Variables included: n	Airwave	ELSA	Generation scotland	Memento
Administration	9	6	7	6	9
Sociodemographic	7	6	7	7	6
Early life environment	4	0	4	0	0
Medical history	20	10	13	10	18
Family disease history	18	0	0	15	12
Psychological status	10	3	7	3	5
Cognitive status	11	4	5	4	6
Lifestyle	7	3	7	2	6
Life functionality	2	0	2	0	2
Physical environment	4	1	2	3	2
Social environment	3	0	3	0	0
Physical examination	7	5	7	5	6
Imaging	6	0	0	0	6
Biosample assays	15	8	8	4	13
Molecular data	1	1	1	1	1
Totals	124	47	73	60	92

**Fig. 1:**
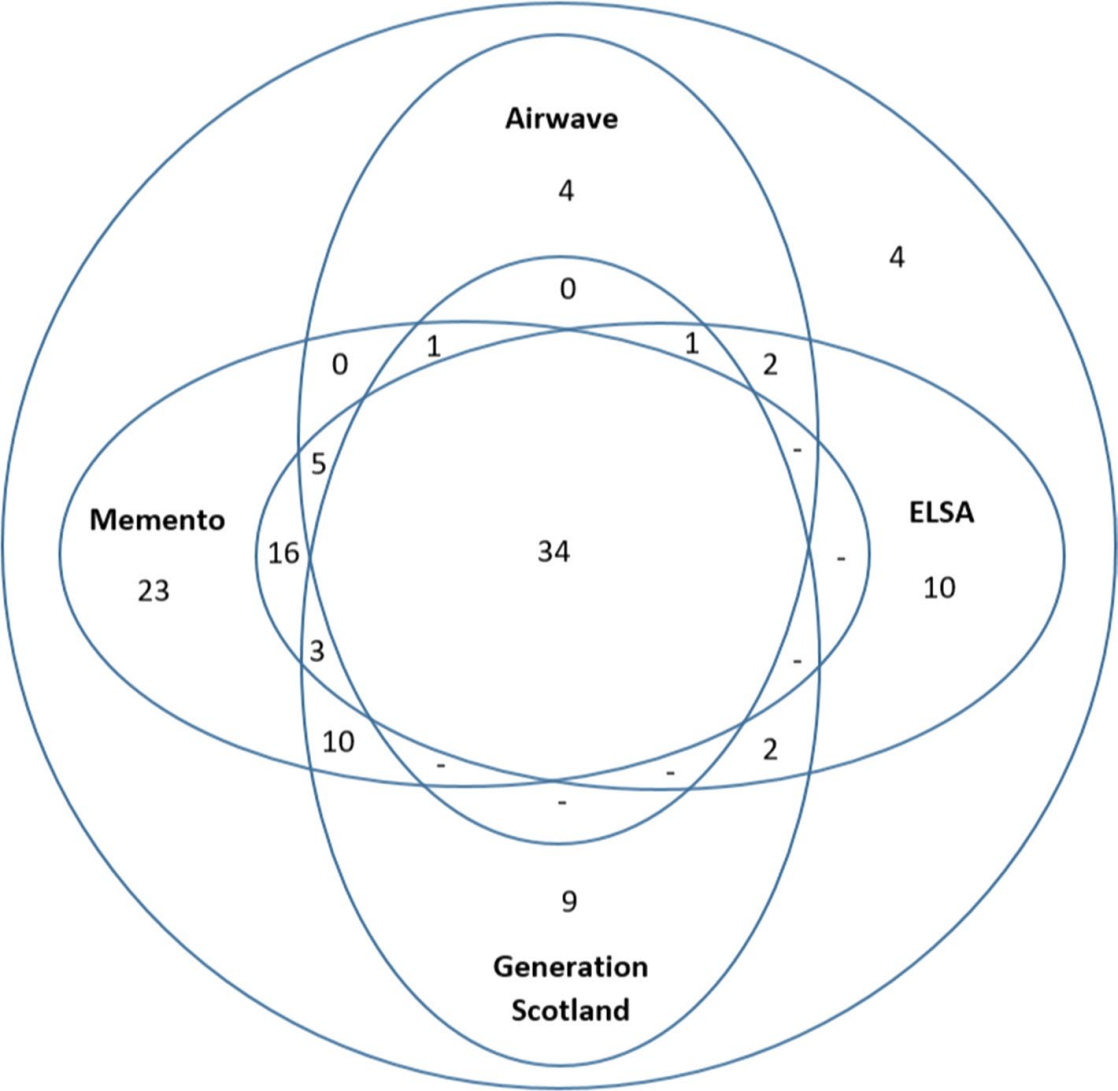
Distribution of core variables across cohorts

The distribution of variables across cohorts also varied, with 34 variables being common to all cohorts, 10 in three cohorts, 30 in two cohorts and 46 in one cohort (Fig. 1). This shows the diversity of the selected cohorts and reflects the range of scientific purpose underlying these datasets. For example, that ELSA and Memento include 13 and 26 unique variables respectively reflects the distinctive scientific foci of these studies; ELSA being focussed on social factors underlying ageing, and Memento focussed more specifically on neurodegeneration.

### Utility

Of the 120 variables that were represented in one or more datasets, 61 (51%) were directly mapped. Direct mapping was generally straightforward but did involve truncation of dates, and the interpretation of text for primary cause of death and medications. For alcohol consumption, although using units per week is translatable with most datasets an ‘other’ option was allowed for when consumption was present but not quantifiable. The harmonisation rules for each variable are shown in the supplementary materials (table S1).

Fifty three (44%) variables were transformed by algorithm. For 44 of these, this was a matter of using a ‘yes/no’ (present/absent) format. For ELSA, the presence or absence of a medical condition was inferred from the data of diagnosis, or a symptom rating score. For Generation Scotland the presence or absence of angina and myocardial infarction was inferred from self-reported heart disease. For Memento several outcomes were indicated as present by interpreting a rating scale score or by a clinical diagnosis. The remaining nine variables required more interpretation (Table 3). For smoking, there was close concordance between the harmonisation rules and the raw data with some interpolation required for ELSA data. This could have been easily addressed by simplifying the scale to a binary ‘ever smoked: yes/no’ format, although this would have been less informative for most of the datasets. Ethnicity was missing in Memento as by law these data are not permitted to be collected in France. Harmonising education was difficult as all the cohorts used qualifications as the index and these varied in detail and across jurisdiction (UK and France). The decision to harmonise on the basis of educational experience rather than qualifications provided a basis for greater integration, although it may be argued that the harmonised scale is less informative. Similarly, for cohabitation and housing type, where simplified scales were applied to the more detailed raw data. For household income local currency was used and aggregated into four quantiles of annual income. For exercise (vigorous, moderate and walking) a simple quantification was not possible due to the diversity of measurement and harmonisation was limited to presence or absence.

**Table 3:** Application of algorithmic transformations across cohorts

Variable	Transformation	Cohort			
		Airwave	ELSA	Generation Scotland	Memento
Smoking	Never	Never	Ever smoked Yes/no	Never	Never
	Ex	Ex	–	Ex	Ex
	current	current	–	current	current
Smoking?	Yes				
	no				
Ethnicity	White	White	White	White	–
	Black	Other	Non white	–	–
	Asian	-	–	Asian	–
	Mixed	-	–	Mixed	–
Education	Post grad equivalent (ISCED 7/8)*	Post grad	–	–	Higher dipl
	Degree equivalent (ISCED 4–6)	Deg. equiv	Deg. Equiv	College/Uni	Degree
	Secondary (ISCED 2/3)	–	Higher ed	–	General Bac
		A level NVQ3	NVQ3	Highers	Tech Bac
		GCSE NVQ2	NVQ2	Standards	CAP/BEP
		NVQ1	NVQ1	CSE equivalent	Elementary
		–	Foreign/other	–	
	< = Primary (ISCED 1)	–	–	Certificate	Primary
		No qualification	No qualification	No qualification	No qualification
Cohabitation	Single	Single	Single	Are you living as a couple? yes/no	Single
	Married/cohabiting	Married	Married	–	Married/cohabiting
		–	Remarried	–	–
		cohabiting	–	–	–
	Divorced/separated	Divorced	Divorced	–	Divorced/separated
		Separated	Separated	–	–
	widowed	–	Widowed	–	Widowed
	Other	Other	–	–	–
Housing type	House/bungalow	–	–	House/bungalow	Single family dwelling
	Apartment	–	–	Apartment/flat	Apartment
	Sheltered/residential	–	–	Hostel	Residential
		–	–	Mobile/caravan	Sheltered
		–	–	Sheltered	Religious community
		–	–	Homeless	Care home
	Other	–	–	Other	Other
Household income	Four quantiles using local currency	Annual: < £25,999, 26,000–37,999, 38,000–59,999, 600,000 +	Gross monthly and annual in Pounds Sterling	–	Monthly: €400– < 800 800– < 1200 1200– < 1800 1800– < 2500 2500 < 4000 4000– < 6000 6000 +
Vigorous exercise	Yes/No	–	Do you attend sports clubs, gym, exercise classes?	–	Days per week Hours per day Minutes per day No vigorous exercise
Moderate exercise	Yes/No	–	–	–	Days per week Hours per day Minutes per day No moderate exercise
Walking	Yes/No	–	–	–	Days per week Hours per day Minutes per day No walking

^*^ISCED International standard classification of education [28]

The six cognitive performance scores were standardised to the Z distribution. The distributions for immediate recall (skew = − 0.42), delayed recall (skew =  − 0.42), digit symbol substitution (skew = − 0.11), verbal fluency (skew = 0.31), were sufficiently Gaussian for Z-scores to be meaningful. For choice reaction time (skew = 1.09), a log_e_ normalisation was used before transforming to z-scores. For this exercise, fluid intelligence is an interpretation of the ELSA numeracy score from ELSA (skew = − 0.54).

## Discussion

For a set of 124 variables, selected for relevance to neurodegeneration, a harmonisation schema designed for data discovery, was applied to data from four diverse population cohorts. Of the 120 variables that were found in the datasets, correspondence between the harmonised data schema and cohort-specific data models was complete or close for 111 (93%). For the remainder, harmonisation was possible with a marginal a loss of granularity. Overall, this demonstrated the feasibility and utility of using relatively simple harmonisation procedures for the purpose of data discovery.

Although these findings indicate value for data discovery, harmonisation is not an exact science and we have not described a mature process. The selection of variables, relevant to neurodegeneration was a reasonable starting point as it framed a specific use-case. Undoubtedly, the selection of specific variables reflected the research interest of the DPUK scientific community and the ADDI Data Harmonisation Group. However, a broad range of variables of generic interest were represented. From this limited variable-set a strong case can be made for incrementally expanding the range of harmonised variables. For a small number of variables (n = 9) using less granular harmonisation rules would have increased inclusiveness. Offering a selection of rules for investigators to choose which best suits their purpose would be straightforward.

Our model was developed using only four cohorts and not all cohorts had data on all variables. Given the use of relatively generic harmonisation rules, the addition of further cohorts with different patterns of ‘missingness’ is unlikely to materially affect the schema as described, but would inform its extension to other variables. For many variables the ‘Yes/No’ indicator was sufficiently generic that whether these variables should be construed as directly mapped or algorithmically transformed is moot. For the processing of free text, the manual interpretation of free text data used here is not scalable or necessarily consistent; the potential of natural language processing for rapid and consistent textual interpretation should be explored. The availability of biosamples was included in the variable list. Technically these are metadata, but were considered informative for data discovery. For cognitive performance, although the harmonisation process was straightforward, without claiming aetiological commonality, grouping tests according to widely used cognitive domains was judged a pragmatic solution. Harmonisation was not applied to longitudinal data. This was intentional to simplify the problem. However, the inclusive and generic nature of the harmonisation schema suggest that applying it longitudinally would be relatively straightforward.

The value of efficient data discovery is commensurate with growth in 3rd party data access and data complexity. Whilst national projects such as the ‘All of Us’ [23] and ‘UK Biobank’ [24] studies are specifically designed for 3rd party access, data discovery in most cohort studies remains challenging. These studies are not resourced to standardise their data, and consensus around how this may be achieved has not been reached. Data platforms, which provide global 3rd party access across multiple datasets provide an opportunity to develop these solutions, as they are positioned to develop harmonisation pipelines that can be applied systematically and consistently across datasets; enabling discovery at-scale and pace.

Efficient data discovery does not just require harmonisation. It also requires tools that exploit the potential that harmonisation brings. Existing cohort-based data discovery tools range from access to rudimentary spreadsheets, through online data dictionaries [25], to more sophisticated ‘shopping basket’ approaches offering discovery and selection [26]. The wide range of functionality and complexity of these tools is a strong argument for the development of tools that simplify discovery across datasets, and that follow-though into data selection. Using data platforms to consolidate approaches to data discovery and variable selection across multiple datasets incentivises the development of more ergonomic and powerful tooling.

The case for streamlining and standardising data discovery can be difficult to make. However, an example of where this has been transformative is the introduction of reference SNP cluster ID (rs) numbers [27]. By establishing rules around how to annotate genetic data, confidence in the provenance of data is increased, transaction costs of data discovery and access lowered, and rigour improved. The point being that simple solutions can be used to increase scientific opportunity rather than restrict academic freedom. This paper demonstrates that a similar exercise for cohort data is technically feasible and argues that it would be highly valuable. The cohorts who participated in this project have all deposited their data with DPUK and are available upon application through DPUK. The harmonised dataset used for this project will be available as an optional data format in the future, integrated within the DPUK curation programme [11].

## Supplementary Information

Below is the link to the electronic supplementary material.

Supplementary file1 (DOCX 62 KB)

Supplementary file2 (DOCX 14 KB)

## Data Availability

Not applicable.
