# Supplementary material for: Evaluating the harmonisation potential of diverse cohort datasets
Source: Eur J Epidemiol. 2023 Apr 26;38(6):605–15. doi: 10.1007/s10654-023-00997-3 (PMC10232583; doi:10.1007/s10654-023-00997-3)
Supplement: Supplementary file 1 — Supplementary file1 (DOCX 62 KB) [file 10654_2023_997_MOESM1_ESM.docx]

**Table S1. Description of harmonisation strategy**
