# Supplementary material for: Evaluating the harmonisation potential of diverse cohort datasets
Source: Eur J Epidemiol. 2023 Apr 26;38(6):605–15. doi: 10.1007/s10654-023-00997-3 (PMC10232583; doi:10.1007/s10654-023-00997-3)
Supplement: Supplementary file 2 — Supplementary file2 (DOCX 14 KB) [file 10654_2023_997_MOESM2_ESM.docx]

**S2 Cohort descriptions**

| **Cohort** | **Population size at baseline** | **Description** |
| --- | --- | --- |
| Airwave Health Monitoring Study (Airwave) | 55,000 | The Airwave Study was established to evaluate possible health risks associated with the use of a digital communication system used by the police forces (TETRA) and other emergency services in Great Britain since 2001. It is a long-term observational study following up the health of the police force with respect to TETRA exposure, and ability to monitor both cancer and non-cancer health outcomes. Baseline screening consisted of an enrolment questionnaire and a health screening. |
| The English Longitudinal Study of Ageing (ELSA UK) | 12,099 | ELSA UK was established to collect longitudinal multidisciplinary data from a representative sample of the English population aged 50 to 100 at recruitment. ELSA was developed as a companion study to the Health and Retirement Study (HRS) in the USA and documents the experience of growing old in England in the 21st century. The study collects objective and subjective data relating to health and disability, biological markers of disease, economic circumstance, social participation, networks and well-being. ELSA has also undertaken a Covid-19 study which is a follow-up study based on the sample of the regular ELSA study Waves to collect data from more than 10,000 ELSA participants, all aged 50 years and over, asking them about their experiences of the COVID-19 crisis. The first wave of data collection took place in June/July 2020 and the second wave started on 4th November 2020, and will be completed by mid-December and looks at changes in participants’ experiences as the COVID-19 crisis evolves. |
| Generation Scotland | 21476 (+2484 postal) | Generation Scotland was established to create a large, family-based intensively-phenotyped cohort recruited from the general population across Scotland, as a resource for studying the genetics of health areas of current and projected public health importance. It aims to identify genetic variants accounting for variation in levels of quantitative traits underlying the major common complex diseases (such as cardiovascular disease, cognitive decline, mental illness) in Scotland. DNA and non-identifiable information from this cohort will be made available to researchers in Scotland and international collaborators. Baseline data was collected at a single clinic visit. Longitudinal data is available by linkage to NHS medical records. |
| Memento | 2323 | The Memento cohort was established to understanding the clinical evolution of patients with early signs that may evoke the onset of Alzheimer's or other related diseases. One goal and challenge of Memento is to measure, repeatedly in patients with early signs, a series of biomarkers (MRI of the brain, genetic analyses, molecular PET imaging, CSF extracted by lumbar puncture) and risk factors (lifestyle habits, drug treatments, quality of life) and to monitor the development of their neurocognitive performance in parallel. All of these observations should make it possible to understand which parameters explain why some people become sick and others do not. In the longer term, the knowledge accumulated thanks to the Memento study could make it possible to diagnose those with dementia at an earlier stage and to offer them, where available, treatments which will make it possible to stop or slow down the evolution of the disease. |
